# Non-native speakers of English or ChatGPT: Who thinks better?

**DOI:** 10.12688/f1000research.161306.2

**Published:** 2025-03-31

**Authors:** Mohammed Q. Shormani

**Affiliations:** 1 Ibb University, Ibb, Ibb Governorate, Yemen

**Keywords:** Center-embedding, generative linguistics enterprise, non-native speakers of English, Large Language Models, ChatGPT, competence

## Abstract

**Background:**

This study aimed to answer the following major question: Who thinks better, non-native speakers of English or ChatGPT?. It provides evidence from processing and interpreting center-embedding English constructions that the human brain surpasses ChatGPT and that ChatGPT cannot be regarded as a theory of language.

**Methods:**

Fifteen non-native English speakers were recruited as participants. A center-embedding English sentence was presented to both the study participants and the ChatGPT. The ability of the ChatGPT to predict and remember was also tested.

**Results:**

The study findings reveal that the human brain is still far ahead of Large Language Models, specifically ChatGPT, even in the case of non-native speakers of L2 English. They also showed ChatGPT’s inability to predict and remember.

**Conclusions:**

The study concludes that the human brain’s ability to process and interpret natural language data and to predict and remember is unique and that ChatGPT still lags behind this unique human ability.

## 1. Introduction

Center-embedding, as in (1), is a syntactic phenomenon in which a matrix clause contains several other relative (embedded) clauses. Put differently, center-embedding occurs when clauses are nested within other clauses, creating significant demands on working memory and sentence processing.

(1) a. The man that the soldier that the thief slapped deceived died.

  b. Men women children dogs bite like marry hate pets.

(1a) presents a triple center-embedding structure, and (1b) a quadrilateral one ((1b) is taken from Karlsson, 2007, p. 8, see also Frazier & Rayner, 1988). These constructions present considerable difficulties for humans because of the complexity of their structures. These center-embedding structures have been investigated since Chomsky and Miller (1963). They impose difficulties on human working memory, giving rise to processing difficulty, perhaps due to the memory load placed on the Faculty of Language (FL) while processing them. Additionally, (1b) is more difficult than (1a), and this difficulty lies in involving more embedded clauses, indefinite nouns/subjects, and the absence of relative pronouns such as *that.* Because of the absence of relative clauses, sentences like (1b) are said to be reduced relative clauses ( Shormani, 2013).

Artificial intelligence (AI) has aroused much controversy among linguists and AI specialists over the use of AI models, such as ChatGPT, and their capabilities. Recently, much debate has been ongoing in this regard. On one extreme, there are scholars who view AI models as incredibly able to perform processing tasks like humans (see e.g., Piantadosi, 2023; Ambridge & Blything, 2024). For instance, Piantadosi (2023) claims that Large Language Models (LLMs) like ChatGPT are good language theories, and they can even “refute” Chomsky’s generative approaches. On the other hand, several scholars refute this claim and argue that, although these models perform great tasks, AI models still fall short of reaching the human brain state ( Katz, 2012; Shormani, 2024a & c). Some scholars provide good evidence from natural language processing tasks that LLMs, including ChatGPT, cannot be considered language theories because they still lag behind the human brain state (see e.g., Zhong et al., 2023; Katzir, 2023).

The first idea behind the inception of AI was how to implement “human intelligence” in computers, making them think like humans. AI is defined as “making a machine behave in ways that would be called intelligent if a human were so behaving” ( McCarthy et al., 1955, p. 11). It is a computer’s underlying ability “to interpret external data correctly, to learn from such data, and to use those learnings to achieve specific goals and tasks through flexible adaptation’ ( Haenlein & Kaplan, 2019, p. 5). The first definition ensues from scientific questions imposed in the 1950s, perhaps with Turing’s (1950) stimulating question “Can machine think?”. The second definition pertains to the modern use of LLMs, such as ChatGPT, in processing, computing, and interpreting natural language data.

The term “artificial” in “artificial intelligence” implies that LLMs’ “intelligence” is not real, and that they do not think like humans (see also Haenlein & Kaplan, 2019). However, there are scholars ( Piantadosi, 2023; Ambridge & Blything, 2024) who see that these models think or process language data even better than humans. Thus, in this study, we aim to determine the extent to which this is true by examining ChatGPT’s ability to process center-embedding English sentences. We also aim to find out who is better at processing these constructions our participants, non-native speakers of English (NNSs), or ChatGPT. Our study recruited 15 NNSs; they are male and female. They are advanced learners of English as a Second Language. Fourteen of them were in the stage of writing their MA and PhD Theses, and one was a level-four student. We asked them a question involving a center-embedding structure. The same question was asked to ChatGPT.

The remainder of this paper is organized as follows. Section 2 briefly presents the Language Faculty and center-embedding structures along with their salient syntactic properties. Section 3 discusses the current situation of LLMs and center-embedding, tackling studies in which AI specialists develop neural algorithms in LLMs to understand center-embedding structures, and studies examining these models’ abilities to understand, process, and interpret such structures. Section 4 describes the methods used in this study. Section 5 presents and discusses the results. Section 6 outlines ChatGPT’s inability to predict or remember things. Section 7 concludes the paper and provides some limitations and suggestions for future research.

## 2. Language faculty and center-embedding

Generative enterprise has adopted a biological and cognitive approach to the study of language, language faculty, and language acquisition ( Chomsky, 1995; Jenkins, 2000; Hauser et al., 2002; Shormani, 2016, 2017). For example, Hauser et al. (2002) discuss the unique features of human language and its evolution, proposing a framework for understanding the language faculty, the organ responsible for language production, and perception in terms of biological and cognitive mechanisms. They argued that there are two states of the Faculty of Language in the Broad Sense (FLB) and the Faculty of Language in the Narrow Sense (FLN) and that there are identified differences between them. FLB includes a combination of systems that support language: i) the sensory-motor system, which is responsible for speech and auditory processing; ii) the conceptual-intentional system, which is involved in meaning and intention; and iii) recursion, which allows the generation of infinite combinations of expressions from a finite set of elements (cf. Chomsky, 2005). In other words, FLN refers specifically to the core computational mechanism for recursion, which, they argue, is unique to humans. This mechanism enables the generation of hierarchical structures, including embedding clauses within matrix clauses. It is also central to human linguistic capability. Hauser et al. (2002) emphasized the interdisciplinary nature of studying language evolution, involving fields such as linguistics, evolutionary biology, and neuroscience. They focused on examining how FLB components evolved independently, exploring possible evolutionary origins and genetic basis of FLN. Between these two states, they suggest that LF sometimes fails to perform some linguistic tasks, including comprehending complex structures, mainly due to the load placed on it or its working memory. FL is, in principle, an intact organ that is genetically wired in human genes, and humans and only humans possess it.

Given the limited ability of FL and working memory in processing complex structures, humans encounter some difficulty in processing center-embedding structures because their syntactically complex structure imposes difficulties for human working memory. Center-embedding has been investigated since Chomsky and Miller (1963) and has been developed in several works. For example, Frazier (1985) found that the processing of these structures was broken down. Dickey (1995) conducted a study in which reading time experiments were presented, which revealed that inserting an ungrammatical resumptive pronoun in the second of the three noun gaps led to faster reading times. Thomas (1995) investigated the cognitive processes involved in understanding sentences with center-embedding and self-embedding structures. These are types of recursive sentence formation in which clauses are nested within one another, creating complex hierarchical structures. He found that center-embedding involves the insertion of subordinate clauses into a main clause. Self-embedding, on the other hand, occurs when multiple embeddings of the same type are nested within each other, leading to more complex and often harder-to-process sentences. He also explored why deeply embedded sentences challenge human working memory and comprehension. Memory limitations and working memory capacity are key factors that make these structures difficult to process (see also Uehara & Bradley, 2002).

Karlsson (2007) studied constraints on multiple center-embedding sentences and their syntactic peculiarities. He examines why sentences with multiple center-embedded clauses like (1) are challenging to process for humans, despite being grammatically correct. In this study, Karlsson introduced the concept of *center-embedding ceiling*, where human cognition struggles to parse sentences with more than two levels of embedding. He further argues that the difficulty arises not merely from cognitive limitations but also from structural constraints inherent to language, providing cross-linguistic examples. He argues that even though multiple center-embeddings are theoretically possible, they are rarely found in actual use because of both cognitive and communicative pressures. He concludes that center-embedding imposes limitations on working memory and the principles of processing efficiency.

Karlsson (2010) explored the limitations of human working memory when processing sentences involving complex syntactic structures, such as multiple center-embedded clauses. Karlsson may build on theories such as Syntactic Prediction Locality Theory (see e.g., Gibson, 1998), which suggests that increased distance between syntactic predictions and their resolutions leads to higher memory and integration costs. These costs become particularly problematic in cases of multiple-center-embedding, as in (1). Specifically, triple-center-embedding structures demonstrate significant processing difficulty owing to the high memory demands of maintaining unresolved syntactic predictions while simultaneously introducing new referents and dependencies. Karlsson argues that beyond a certain threshold, such structures exceed the working memory capacity, rendering them ineffective in real-time. Karlsson’s (2010) study concluded that working memory imposes a hard constraint on sentence complexity, highlighting the importance of syntactic simplicity and local dependencies in human language comprehension.

## 3. Center-embedding and LLMs

As stated above, center-embedding is a linguistic construction in which relative clauses (full or reduced) are inserted into the middle of a sentence, creating a highly nested structure. In this section, we focus on LLMs and their involvement in center-embedding phenomena. Center-embedding has been involved in two aspects of natural language processing (NLP), resulting in ample studies. These studies can be classified into two types: i) studies concerning the development of Neural Networking Algorithms (NNAs) with center-embedding algorithms, and ii) studies concerning testing LLMs’ abilities to process and interpret these constructions. Concerning the first type, for instance, Jiang et al. (2023) developed a prompt-based method with explicit one-word limitation (PromptEOL), a method that leverages prompts for embedding sentences, and explores its efficacy both with and without fine-tuning. PromptEOL integrates in-context learning by providing specific prompts to LLMs, allowing them to generate sentence embeddings without additional parameter updates. When fine-tuning is applied, PromptEOL significantly enhances the performance of LLMs in various sentence-level tasks. However, without fine-tuning, PromptEOL surpasses state-of-the-art methods, such as SimCSE, in semantic textual similarity benchmarks. The method benefits from model scaling, with embeddings improving as the model size increases. However, there are a number of challenges: i) diminishing returns: the performance plateau for very large models raises questions about the scalability limits of LLMs in certain tasks, and ii) task-specific optimization: tailored approaches to optimize embeddings for different downstream applications are highlighted. They concluded that their proposal provides a strong case for the scalability and adaptability of LLMs in sentence embedding tasks. By introducing PromptEOL, it provides a pathway to efficiently harness the power of large models, bridging the gap between the raw model size and practical performance.

Harris et al. (2024) explored a method to improve text embedding performance by preprocessing the input text using LLMs, specifically ChatGPT 3.5. They consider this approach crucial for various NLP tasks, but there are a number of factors that limit its functionality, such as vocabulary, lack of context, and grammatical errors. This approach involves enriching text with context, correcting grammatical errors, disambiguating terms, and including relevant metadata before generating embeddings. The aim is to enhance the performance of embedding models in downstream tasks such as classification and clustering. This study evaluated the method on three datasets: Banking 77 Classification, TwitterSemEval 2015, and Amazon Counter-Factual Classification. It uses metrics, such as cosine similarity and accuracy. The results show notable performance improvements, particularly on the TwitterSemEval dataset, where the proposed technique achieved a significant leap from the previous best performance (85.34 vs. 81.52 on the massive text embedding benchmark). However, improvements on other datasets were mixed, underscoring the dependency on the dataset characteristics.

The second type of studies examined LLMs to process and interpret center-embedding structures. For example, Kodner et al. (2023) reply to Piantadosi’s assertion that modern LLMs challenge Chomsky’s linguistic theories. The authors defend the relevance of generative linguistics and argue its continued importance in understanding human language. They focus on four issues: i) The data gap: they emphasize the disparity between the vast data requirements of LLMs and the minimal exposure young children need to acquire language. This highlights the unique mystery of human language acquisition that generative linguistics seeks to explain; ii) artificial vs. natural insights: they draw an analogy between LLMs and airplanes, suggesting that while airplanes reveal much about engineering, they offer little insight into natural avian flight. Similarly, LLMs’ functioning of LLMs may not elucidate the cognitive mechanisms underlying human language; iii) limits of LLMs as scientific theories, and the authors argue that scientific theories require interpretable explanations, not just predictive accuracy. Since LLMs lack explicit theoretical frameworks, they cannot replace linguistic theories; and iv) the necessity of independent linguistic theories: evaluating LLMs’ capabilities still depends on understanding human linguistic capacities. Generative linguistics provides a robust framework for such evaluations, underscoring its indispensable role in linguistic sciences. Kodner et al. concluded that generative linguistics will remain crucial in advancing our understanding of language despite technological progress in computational models.

Dentella et al. (2024) asserted that language is not an attribute that can be ascribed to LLMs. In their experiment, seven LLMs failed to respond to simple questions based on textual input, including examples, such as (2).

(2) a. “John deceived Mary and Lucy was deceived by Mary.”

  b. “In this context, did Mary deceive Lucy?”

While human participants succeeded in this task, although they sometimes err, not only did LLMs fail, but they also kept doing the same errors, which is not human. If a human makes a mistake once, he/she does not repeat it if his/her attention is drawn to that error. They recruited 400 native English speakers, utilizing GPT-3 and GPT-3.5. They concluded that LLMs lack a compositional operator that integrates and regulates the grammatical and semantic information.

Katzir (2023) criticizes the claim that LLMs such as GPT-3 and similar models serve as robust theories of human linguistic cognition. Katzir argues against Piantadosi’s (2023) argument that LLMs outperform generative linguistics in explaining human language cognition. He provides objections in relation to three phenomena: i) Competence vs. performance: Katzir emphasizes that LLMs lack the ability to distinguish between linguistic competence and performance. The former refers to the native speaker’s underlying knowledge of his/her language, whereas the latter refers to his/her use of language in actual situations ( Chomsky, 1965). In Chomsky’s words, there is “a fundamental distinction between competence (the speaker-hearer’s knowledge of his language) and performance (the actual use of language in concrete situations)” (p. 4). In this sense, there is perhaps a distinction between humans and machines or computers in that competence is a human attribute, specifically a characteristic of the human brain whose “linguistic performance” is an indication of the underlying linguistic competence. On the other hand, computers or LLMs, in specific terms, can produce a similar phrase/sentence or even a text based on the data (i.e., the corpus) they have been trained on, which does not entail that they have “competence” like humans (see also Kaufer, 1979). If this is on the right track, then it follows that the distinction between competence and performance is central to understanding human linguistic behavior, as humans often struggle with sentences due to processing limitations, not a lack of competence. In contrast, LLMs’ errors reflect deficits in their statistical learning mechanisms, not resource constraints; ii) likelihood vs. grammaticality: Katzir considers that humans can discern grammatical but unlikely sentences from likely but ungrammatical ones. This is perhaps due to the neurological mechanism the brain working mechanism is based on. In terms of connectionist models of the FL models, the human brain tries to identify the functional task through which it comprehends or produces speech ( Arbib & Caplan 1979; Nelson, 1978; Arbib, 1982), and iii) typological universals: LLMs do not inherently explain cross-linguistic typological universals, which generative linguistics seeks to address. LLMs may be insufficiently biased towards these universals, making them implausible models for understanding human linguistic diversity.

Zhong et al. (2023) evaluated ChatGPT’s natural language understanding (NLU) capabilities against fine-tuned BERT models (BERT-base, BERT-large, RoBERTa-base, and RoBERTa-large) using the GLUE benchmark. They found that ChatGPT’s performance is comparable to that of BERT-base (78.7% vs. 79.2% average score) but lags behind more advanced models such as RoBERTa-large (87.8%) (see also Ettinger, 2020). However, ChatGPT struggles with paraphrase detection and semantic similarity, underperforming BERT-base by as much as 24% in some cases. This study suggests that while ChatGPT is versatile, it still lags behind highly specialized fine-tuned models in specific NLU tasks. This underscores the complementary nature of task-specific fine-tuning and the broad generalization abilities of LLMs, including the ChatGPT. This aspect can also be contrasted with the human brain, a species-specific property with a unique working mechanism (see also Chomsky, 2009; Berwick & Chomsky, 2016).

However, one should also elaborate on studies that see LLMs capable of doing linguistics tasks like human including Piantadosi (2023). To elaborate on Piantadosi’s views, he proclaims that LLMs refute Chomsky’s generative approach to the study of language. He argues that the success of LLMs undermines the necessity of an innate UG. He asserts that LLMs, which acquire linguistic competence through being trained on massive amounts of data rather than preprogrammed grammatical structures, demonstrate that statistical learning and pattern recognition may be sufficient for language acquisition. This perspective aligns with usage-based and emergentist models, which emphasize learning from experience rather than innate rules. However, critics argue that while LLMs can process and generate human-like language, they do so in ways fundamentally different from human cognition (see e.g. Katzir, 2023). Unlike children, who learn language efficiently from limited input, LLMs require immense datasets and lack explicit rule-based interpretability. Additionally, they may exhibit impressive linguistic performance without true competence, that is, they generate coherent language without understanding it in a human-like way.

Another recent study that could be addressed here is Ambridge and Blything (2024). Like Piantadosi (2023), they claim that generative linguistics often relies on verbal accounts that may lack precise quantitative predictions or depend on ancillary assumptions. In contrast, they suggest that LLMs, trained on extensive linguistic data, can effectively learn and represent these verb argument structures, offering more accurate predictions without the need for additional assumptions. This leads them to propose that LLMs might serve as superior models for understanding certain linguistic phenomena compared to traditional theoretical approaches. In their study, they tested LLMs ability to predict verb argument structures, particularly focusing on English causative constructions. They highlight the phenomenon where certain verbs can appear in both intransitive-inchoative as in *The ball rolled* and transitive-causative forms as in *Someone rolled the ball* whereas others are restricted to intransitive forms as in *The man laughed* but not *Someone laughed the man*. However, as has been discussed in details so far, this perspective has sparked debate within the linguistic community. Linguists criticized these perspectives, arguing that while LLMs excel at pattern recognition and prediction based on large datasets, they do not necessarily provide explanatory theories of linguistic competence. For instance, Fox and Katzir (2024) caution against equating the predictive success of LLMs with theoretical understanding, emphasizing the need for models that offer explanatory adequacy beyond empirical data fitting. Similarly, Reiss and Volenec (2024) asserted that adopting LLMs as theories of language overlooks the distinction between modeling linguistic behavior and explicating the underlying cognitive mechanisms. These discussions underscore the ongoing discourse on the role of LLMs in theoretical linguistics, balancing their empirical successes with the quest for deeper explanatory frameworks.

As for non-native speakers’ ability to process syntactically complex structures, for example, Lee and Phillips (2023) investigate why non-native English sometimes outperform native speakers in processing subject-verb agreement, particularly in contexts involving agreement attraction. Agreement attraction occurs when a nearby noun erroneously influences verb agreement, leading to acceptance of ungrammatical sentences. They found that native speakers are more susceptible to such errors, while L2 learners, especially those whose first language lacks number agreement, exhibit greater accuracy. This difference is attributed to native speakers’ reliance on implicit linguistic cues, making them more prone to interference from misleading elements. In contrast, L2 learners depend more on explicit processing strategies, which may enhance their accuracy in these situations. The study suggests that the cognitive mechanisms underlying language processing differ between native and non-native speakers, leading to situations where L2 learners’ analytical strategies result in fewer agreement errors.

Additionally, Wu et al. (2020) studied the syntactic complexity in English faced during academic writing, exploring how syntactic complexity is manifested in academic writing by users of English as a Lingua Franca (ELF). Using a corpus-based approach, the study analyzes sentence structures, clause embedding, and phrasal complexity in ELF writing, often comparing it to native English academic texts. It finds that ELF writers employ varying levels of syntactic complexity, influenced by their linguistic backgrounds, yet still achieve communicative effectiveness. The study challenges the notion that native-like complexity is essential for academic rigor, emphasizing instead the importance of clarity and coherence.

There are also studies concerning enhancing the research and academic writing abilities of non-native speakers of English through LLMs. For example, Li et al. (2024) conducted a study to examine the potential of ChatGPT in enhancing the academic writing skills of NNSs of English medical students. The study involves 27 third-year medical students from Sichuan University, all of whom were tasked with writing a mini-paper in English. After the initial writing, students used ChatGPT over a two-week period to revise and improve their papers. The study aims to assess the AI’s effectiveness in enhancing aspects of writing such as grammar, coherence, and structure. The results indicated significant improvements in these areas, with students reporting that ChatGPT provides valuable real-time feedback and suggestions that helped refine their work. Participants expressed positive feedback about the AI tool, noting that it helped them learn and apply academic writing conventions more effectively. However, the study also emphasized the importance of using AI tools like ChatGPT as a supplement to traditional teaching methods rather than a replacement, encouraging students to critically evaluate AI-generated suggestions. This research suggests that AI, particularly ChatGPT, can play a supportive role in improving academic writing skills for non-native English speakers, especially in complex fields like medical education, and provides a promising direction for integrating AI into educational strategies.

Another study is conducted by Hwang et al. (2023) who explored the potential of LLMs like ChatGPT to assist NNSs of English researchers in academic writing. They suggest that ChatGPT can function as a personal, round-the-clock English tutor, aiding in the composition of challenging sections such as the Introduction and Discussion of research papers. The authors draw an analogy to the mythological gift of fire, proposing that ChatGPT offers a transformative tool for enhancing writing proficiency. However, they also caution about potential over-reliance on AI, emphasizing the importance of critical engagement with AI-generated content to maintain academic integrity and personal skill development. However, Hwang et al. (2023) warn against the over-reliance on AI. They emphasize that while ChatGPT can support language improvement, it should not replace the essential skills that researchers must develop themselves. The critical engagement with the AI’s output is important to ensure academic integrity and to avoid over-reliance, which could lead to a decrease in personal writing ability over time.

Thus, considering the above review, the present study seeks to answer the following questions.

1. Who thinks better, NNSs or ChatGPT?
2. To what extent do NNSs understand, interpret, and perceive center-embedding structures?
3. To what extent do LLMs, specifically ChatGPT, understand, interpret, and perceive center-embedding structures?

## 4. Methods

### 4.1 The study data

The study data consisted of a sentence involving the center-embedding phenomenon, namely, *The man that the soldier that the thief slapped deceived died.* The same sentence was used as an instrument for judgement, that is, we asked ChatGPT to judge whether the sentence was grammatical. We used only one center-embedding sentence as the data of the study for two reasons: i) if human participants err in one sentence, they will commit the same errors in similar sentences, and the same thing can be said about ChatGPT, and ii) to avoid redundancy ensuing from discussing and/or interpreting the same data that contain the same errors either by human participants or ChatGPT.

### 4.2 Participants

This study involved 15 male and female participants. They are NNSs who are advanced L2 English students. Their L1 is Arabic, specifically Yemeni Arabic, and their advanced level is judged by being MA and PhD students, writing their Theses: two are PhD students, doing their PhD Dissertations, twelve are MA students, doing their MA Theses, and one is a level-four student. They have studied English for more than 10 years, 6 years at school and 4 or more than 4 years at university. In their study of English as a second language, they have studied knowledge-base courses including linguistics, literature, translation, in addition to skill-base courses such as Writing, Reading, and Spoken. MA and PhD participants are now writing their Theses, speciating in areas such as Syntax, Language Acquisition, Morphology, and Translation. These participants’ characteristics were our criteria of recruitment. These characteristics we believe meet the syntactic complexity of center-embedding structures. Additionally, we focused on a homogeneous group of advanced non-native speakers of English to ensure controlled investigation, and avoid complexity, hence excluding the possibility of involving a controlled group of native speakers. For NNSs’ responses, see Shormani (2025).

### 4.3 Procedure

The English sentence was sent to the participants via WhatsApp. The question was:

“What each did in the following sentence:

“The man that the soldier that the thief slapped deceived died.”

The man:

The soldier:

The thief:

I am doing a research and I need your answer.”

**Figure 1.**
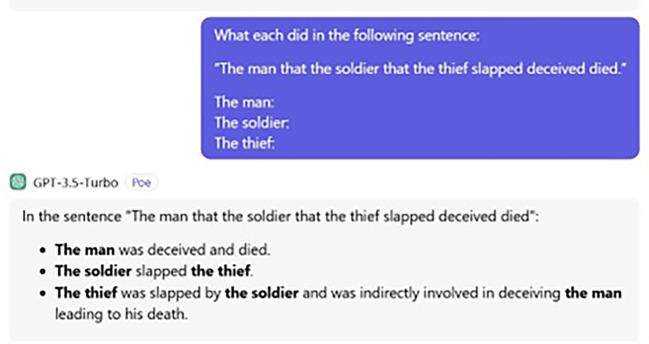
ChatGPT’s processing task.

**Figure 2.**
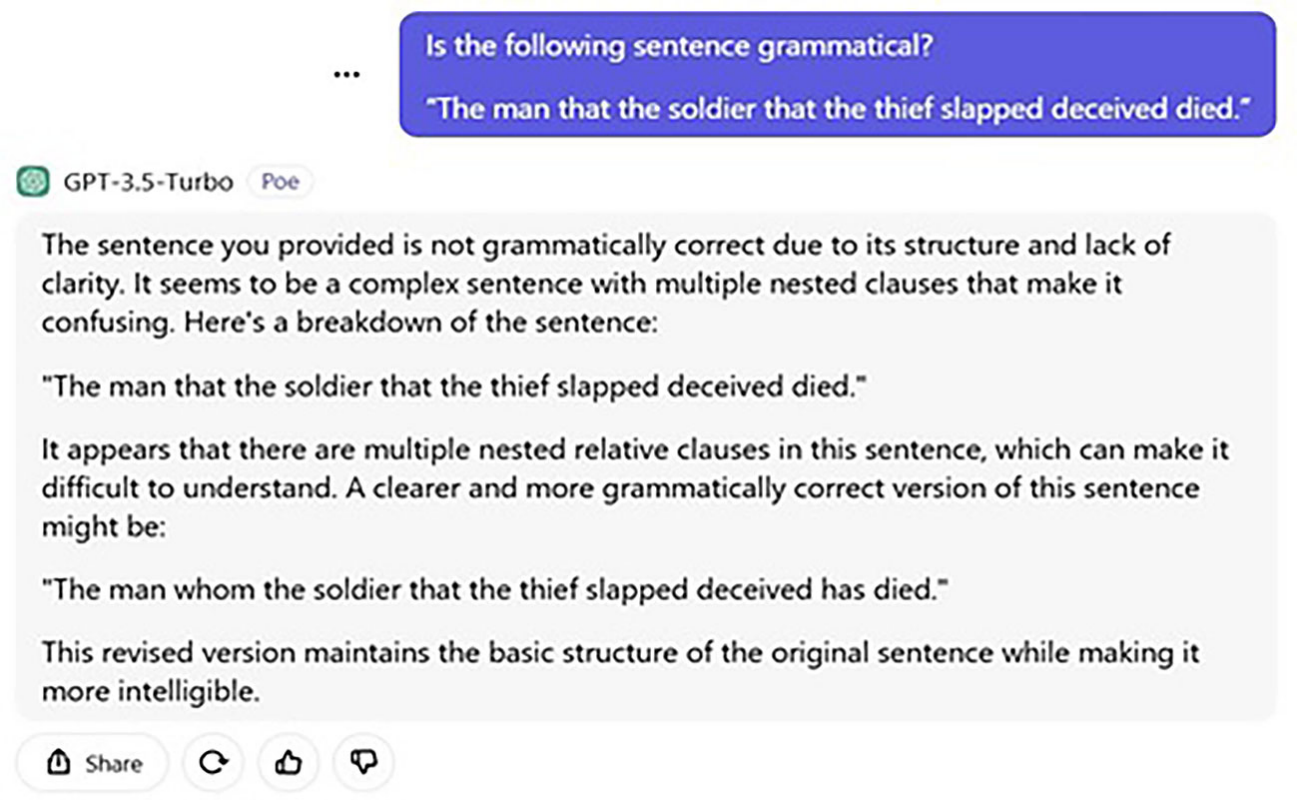
ChatGPT’s judgement task.

The participants were given 24 h to answer the question. The study question was sent to the participants on November 7, 2024. The time they submitted their answers was somehow different for each one; some answered immediately, some took about two hours, and two submitted their answers the next day, but within the time allotted. Those who took a long time did not see our message. We did not respond to some of them asking for further contextual clues. Our response to them was “We cannot give you any further clarification, because doing so will affect the study results.” They were, in fact, instructed to answer the study question individually. They were also explicitly informed that their responses should reflect their own understanding of the center-embedding sentence. To refer to the participants, we did not use their real names; instead, we used numbers (1-15). The same question was asked to ChatGPT. We utilized the ChatGPT-3.5, because it is the only available free version for us during conducting the study. ChatGPT’s answer was recorded on October 3, 2024. The ChatGPT version was accessed through the official ChatGPT client, which ensured that the model used was the standard version available to us at the time of the study. Figures 1 and 2 display the ChatGPT’s answers for both the processing and judgement tasks.

### 4.4 Methods of analysis

The study adopts a quantitative and qualitative approach to the analysis of the data obtained, employing descriptive and comparative methods. The quantitative approach lies in providing statistical metrics (i.e. frequency and percentage) of the results of both human and ChatGPT answers. Human participants’ responses are divided into: i) correct responses, ii) partially correct responses, and iii) incorrect responses (categorized by error type). The same procedure was applied to ChatGPT’s responses. The analysis includes syntax, i.e. grammaticality, Semantics, i.e. interpretations and comprehension failure, mainly in ChatGPT’s inability to reconstruct sentence meaning correctly. On the other hand, quantitative analysis focuses on analyzing and describing the quantitative results accounting for these results through linking to theoretical aspects of generative approach to linguistics and L2 acquisition. It also focuses on identifying error patterns of recurring linguistic challenges in human and ChatGPT responses. The comparative method consists in comparing and evaluating both human and ChatGPT responses amalgamating the comparison and evaluation with AI and generative linguistics interpretation, thus involving a structured comparison and evaluation and reflecting a systematically coded scheme.

## 5. Results and discussion

### 5.1 Results

In this section, we tabulate the NNSs and ChatGPT responses. Table 1 lists these responses. It displays the answers of 15 participants for the three entities: *the man, the soldier* and *the thief.* It also presents ChatGPT’s responses to these entities.

**Table 1.** NNSs’ and ChatGPT’s responses (for *the man, the soldier and the thief* ).

Participant	The man	The soldier	The thief
1	died	deceived	slapped
2	died	slapped	slapped
3	died	deceived	slapped
4	died	deceived	slapped
5	died	deceived	slapped
6	died	deceived	slapped
7	slapped	deceived	died
8	nil	nil	nil
9	died	slapped	deceived
10	died	deceived	slapped
11	died	deceived	slapped & deceived
12	nil	nil	nil
13	died	deceived	slapped
14	died	deceived	slapped
15	died	deceived	slapped
ChatGPT	died	slapped	deceived

### 5.2 Discussion

As Table 1 shows, almost all the participants made good judgements on the stimulus sentence and stated what *the man, the soldier,* and *the thief* each did, while ChatGPT failed to do so. We present only those who got it wrong. Participant 2 made two mistakes, viz. s/he did not get the correct answer for the *soldier* and *thief.* Participant 7 got it right for the soldier, but s/he mixed between the *man* and *thief.* Participant 9 did it right for the *man*, but s/he mixed what the *solider* and *thief* did. Participants 8 and 12 left them in an undone state. However, we consider this as if they were wrong. Thus, we have 12 incorrect answers out of the 45 total answers for the three entities, that is, *man, soldier* and *thief.* In human answers, 26.7% were incorrect and 73.3% were correct. Five participants (including those who left it undone) made mistakes, that is, 33.3% (10 participants, viz. 66.7%) provided correct answers. ChatGPT provided only one correct answer, that is, only for the *man.*

The way ChatGPT answers the question is strange because it adds information messing. Human participants also add more information; for instance, stating the object who receives the action, but they do not mess things. Put simply, although the question was clear that what is needed is only what each subject did, no matter what action the object received, humans added reasonable information, while ChatGPT provided nonsensical information. That is, ChatGPT errs even with this (extra) information. The following is ChatGPT’s exact answer:

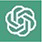
 The man was deceived and died.

  The soldier slapped the thief.

 The thief was slapped by the soldier and indirectly involved in deceiving the man, leading to his death.

**Figure 3.**
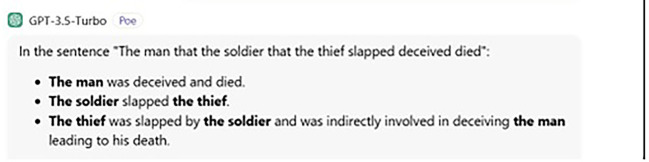
ChatGPT’s answer to judgement question.

In ChatGPT’s answer, we find that it does not understand center-embedding structures, although it has been developed for this purpose, as we have seen so far. In other words, ChatGPT has incorporated algorithms to process center-embedding sentences (see e.g., Jiang et al., 2023; Harris et al., 2024; and references therein), but it fails to process and interpret sentences. In *The man was deceived and died,* we know that *the man* died, but he deceived no one. Additionally, ChatGPT’s answer with regard to the *soldier*, that is, *The soldier slapped the thief* is completely incorrect. The third part of the answer was nonsense. This is further shown in Figure 3 below.

Additionally, ChatGPT’s failure to answer this question urges us to examine its grammaticality judgement. Put simply, if ChatGPT’s answer were as such, one may wonder what and how its grammaticality judgement is. We asked the following question to determine its judgement in terms of grammaticality.

User: Is the following sentence grammatical?

“The man that the soldier that the thief slapped deceived died.”

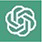
 The sentence you have provided is not grammatically correct because of its structure and lack of clarity. It seems to be a complex sentence with multiple nested clauses that makes it confusing. Here, is a breakdown of the sentence.

  “The man that the soldier that the thief slapped deceived died.”

  It appears that there are multiple nested relative clauses in this sentence, which can be difficult to understand. A clearer and more grammatically correct version of this sentence is as follows.

  “The man whom the soldier that the thief slapped deceived has died.”

The revised version maintains the basic structure of the original sentence while making it more intelligible.

**Figure 4.**
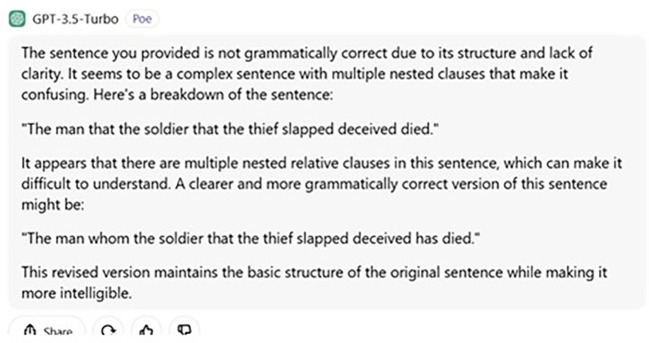
ChatGPT’s answer to judgement question.

This is also schematized in Figure 4 below:

This finding makes our study partly contrast with Katzir (2023): while ChatGPT judges Katzir’s sentence as “grammatically correct,” in our study, it judges the sentence as “not grammatically correct.” This also provides another piece of evidence that ChatGPT is inconsistent. However, all NNSs considered this grammatical. Although we have not asked them directly about it, we, in fact, inferred their grammaticality judgement indirectly, because none of them said the sentence is “wrong/ungrammatical”. Regarding ChatGPT’s answer in relation to misunderstanding center-embedding structures, our study is in line with Katzir’s (2023) findings. ChatGPT in both studies lagged behind the expected performance. Both studies demarcate its poor performance, which requires further specialized development. Our study also supports Dentella et al.’s (2024) study, in which human participants outperformed ChatGPT. The difference between our study and theirs concerns the type of participants - while our participants were NNSs, their participants were native speakers of English.

The fact that LLMs, such as ChatGPT, fail to process center-embedding structures could be accounted for if we know the basis on which they function. They depend largely on statistics and statistical procedures. Put differently, LLMs working mechanism is based on probabilities, i.e. they just “guess” or “predict” the n-gram word, which is not always error-free. This deficiency in their mechanism could be attributed to their “competence,” viz., their underlying ability to point out or choose the correct word, and not predict or guess it. LLMs may also lack “competence” in the technical linguistic sense. In contrast, native speakers of an L may fail to perform a linguistic task, as in the case of Dentella et al. (2024), and their failure is not ascribed to “deficiency” in their competence but to performance, which is particularly ascribed to psychological factors such as slips of the tongue, fatigue, or not paying attention, which are all nonlinguistic (see also Chomsky, 2009). Likewise, if we assume that NNSs have built a “perfect” linguistic system, i.e. they mastered the L2 linguistic system, and if they fail to do a linguistic task, this failure cannot be ascribed to a “deficiency” in their linguistic competence, but rather to their performance, that is, their ability to use or judge a piece of language. Additionally, NNSs have been reported to outperform native speakers, specifically in agreement phenomena ( Lee & Phillips, 2023).

These facts have long been observed in generative enterprise. For instance, Chomsky (1975) points out that the generative approach to the study of language “contrasts with a statistical approach that leads to an ordering of sequences from more to less probable, rather than a sharp division into two classes within which no such gradations are marked.” His famous nonsense phrase is a good case-in-point here. The sentence *Colorless green ideas sleep furiously* and its opposite sequence *furiously sleep ideas green colorless* as described by him “are not distinguished by their assigned probabilities. If probability is to be based on an estimate of frequency in some English corpus, then this probability will be so in both cases” (p. 145). Additionally, corpus-based probabilities and their low order lack explanatory adequacy due to their being “predictional” like when, for instance, a sentence appears in a linguistic corpus ( Kaufer, 1979). We can conclude that our analysis supports studies that critique LLMs, in that LLMs, though powerful tools, fail to account for the dynamic interplay between competence and performance, which is central to human linguistic behavior (see also Katzir, 2023).

## 6. ChatGPT’s wrong prediction and remembrance

**Figure 5.**
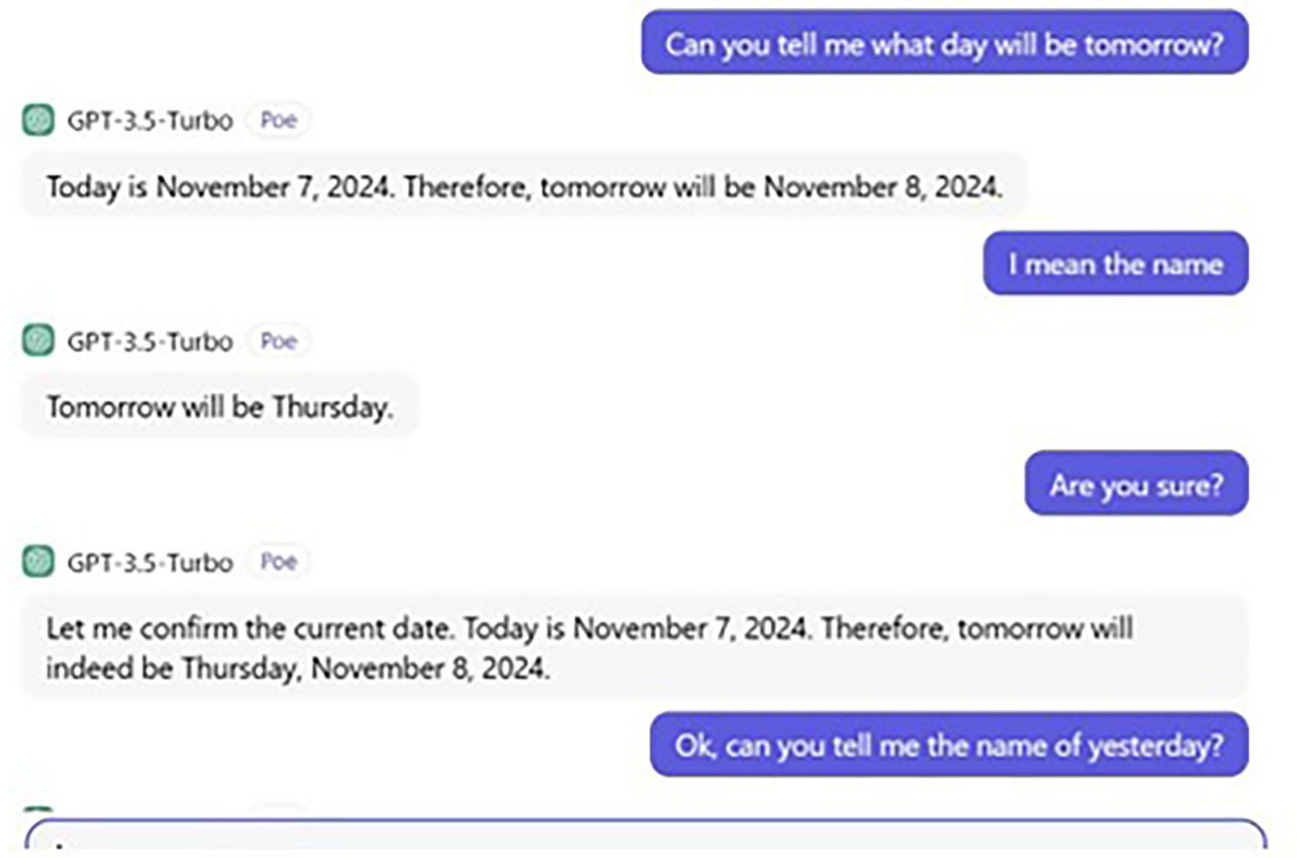
ChatGPT’s wrong prediction.

**Figure 6.**
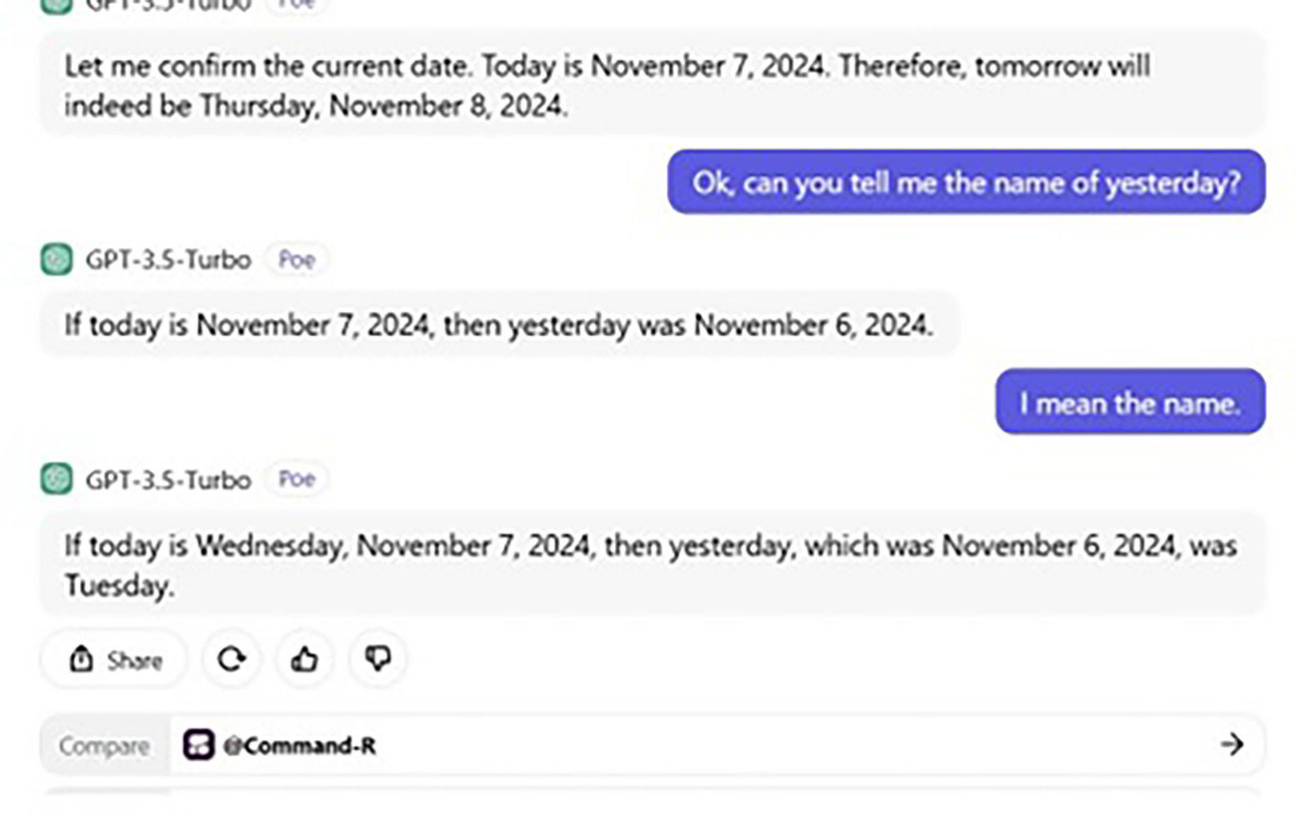
ChatGPT’s wrong remembrance.

From a curious perspective, a further conversation takes place between us and ChatGPT the topic of which was to tell us the date and day of “Tomorrow” and “Yesterday.” The date and day of our conversation were Thursday and 7, 2024, respectively. Our whole conversation with ChatGPT is demonstrated in Figures 5 and 6.

When we asked ChatGPT to tell us Tomorrow’s name, it gave us a date. If today is Thursday 7, 2024, tomorrow, then is Friday 8. Although the date was correct, ChatGPT failed to predict the name of *tomorrow*, giving us *Thursday* instead of *Friday.* Then its ability to remember is also questionable. Put simply, ChatGPT also fails to give us the name of *yesterday*, providing *Tuesday* instead of *Wednesday* (see also Shormani, 2025). However, if we had asked humans (say, a participant of ours), they would have certainly given us the correct answer. In fact, in this very aspect, Google seems to be better than ChatGPT, and we asked Google to tell us the date and (name of ) day of both *tomorrow* and *yesterday*, and it provided the correct answer. Thus, this conversation gives us a clear clue that ChatGPT’s ability to predict is still far from the human ability to predict and remember. This also supports our argument that ChatGPT still lags behind the human brain state and that humans possess a unique ability not only to process and comprehend linguistic data, but also any mental process required.

## 7. Conclusions and limitations

To conclude, we examined the ability of both NNSs and ChatGPT to process and interpret center-embedded English sentences. We recruited 15 NNSs as participants: advanced L2 English students, and BA, MA, and PhD students. They outperformed ChatGPT in both processing/interpreting and judging the grammaticality of a given center-embedding sentence (cf. also Lee & Phillips, 2023). Thus, our study highlights the fact that the human brain of (advanced) NNSs, like that of native speakers of English (as in the case of Dentella et al., 2024), processes and interprets a complex English structure, viz., a center-embedding sentence far better than LLMs such as ChatGPT, and that these LLMs cannot be considered powerful theories of language, as recently claimed by some researchers ( Piantadosi, 2023; Ambridge & Blything, 2024). Another conclusion that can be drawn here concerns NNSs and their linguistic competence. Given the percentage of their answers to the center-embedding sentence presented to them (73.3%), we can conclude that their linguistic system of English is similar to that of native speakers. Those who erred (including those who did not provide answers) if we assume that NNSs have built a “perfect” linguistic system, i.e. they mastered the L2 linguistic system, and if they fail to do a linguistic task, this failure cannot be ascribed to a “deficiency” in their linguistic competence, but rather to their performance, i.e. their ability to use or judge a piece of language.

The study findings revealed that NNSs performed better than ChatGPT, which indicates that: i) LLMs need further development, and ii) the human brain (even in the case of NNSs) surpasses LLMs. Although center-embedding imposes difficulties for native speakers’ FL working memory (see e.g., Dickey 1995), the fact that our participants, who are NNSs, performed better than ChatGPT has several implications for both generative linguistics enterprise and AI technology, ChatGPT, in particular, the most prominent of which are: i) generative linguistics enterprise: NNSs’ competence mirrors that of native speakers. This, in turn, indicates that once a learner develops and masters the linguistic system of an L2, English, this linguistic system does not differ much from that of the native speaker of this L2 (see e.g., Cook, 1983; White, 2003; Shormani 2014a & b, 2015, 2016, 2023). This is further evidence of Chomsky’s conceptions of Language Faculty, Universal Grammar, Genetic Endowment, and Language Innateness ( Chomsky, 1957, 2001, 2008, 2021; see also Shormani, 2016, 2023, 2024b & c), ii) AI technology: AI LLMs still require further development to overcome these challenges. The study also refutes Piantadosi’s (2023) arguments that LLMs are good theories of language, and that these models cannot refute Chomsky’s generative enterprise. This also supports Dentella et al.’s (2024) findings that language is an attribute of humans and only humans, a species-specific property, and that it cannot be attributed to LLMs.

The study findings also provide insights into LLMs’ working mechanisms of LLMs. In our study, the ChatGPT errs in both the processing and judgement tasks. However, this does not seem strange given the fact that LLMs base their predictions purely on statistical likelihood, often favoring plausible continuations that may be grammatically incorrect, which demonstrates a fundamental limitation compared to human cognition (see also Katzir, 2023). While LLMs may be used as engineering tools, their design and functionality fall short of providing a scientific model for human linguistic cognition. Our aim in this study is to pinpoint the actual State of Human brain (even in NNSs) and that of LLMs. The stimulus was a center-embedding sentence. This sentence is used to highlight the fundamental differences between human linguistic cognition and capabilities of LLMs, which are basically statistical. The fact that humans often struggle with center-embedded sentences like (1) above, cannot be ascribed to a deficiency in their genetic linguistic knowledge, but it is simply due to the fact that these structures tax working memory, and the more the center-embedding sentence gets complex, the more the load is placed on FL. Therefore, human failures are attributed to performance limitations, rather than a lack of understanding of syntactic rules (or competence) (see also Shormani, 2012). However, the success or failure is based on their statistical training and inherent model structure. Their competence directly reflects their behavior; errors are not due to transient resource constraints, but due to the limitations of their learned representations ( Katzir, 2023). According to Katzir, although humans may initially struggle with center-embedded sentences, they can often parse them correctly with additional time or contextual clues. Adaptability is a hallmark of human cognition and is absent in LLMs. LLMs lack resource-based recovery mechanisms. The ability of LLMs to distinguish between competence and performance limits their usefulness as models of human cognition; hence, they cannot be regarded as powerful language theories (cf. Shormani, 2024b & c). These findings are in line with those of Katzir (2023). Like Katzir, we utilized center- embedding to examine whether LLMs can capture the nuances of how humans process, interpret, and judge complex linguistic structures.

However, this study has some limitations. The first limitation concerns the type of sentence involved. A comprehensive study might involve other types of complex English sentences such as those involving DP islands, multiple wh-questions, anaphora, and weak/strong crossovers to assess both NNSs’ and ChatGPT’s capabilities in a wider context. The second important limitation that can be tackled here is the version of ChatGPT, viz., -3-5. A broader study could utilize ChatGPT-4. The latter is said to be more developed in functionality and features, and utilizing it could widen the scope and purpose of a further study.

## Ethics and consent

All participants provided consent to publish and replicate their data. This was through a WahtsApp message which means: “Please note that participating in this study is voluntary, and that your data will be made available for publishing and replicating, if needed.” The study was also approved by the Department of English Studies, Ibb University, Ethical Body in such cases (the ethical approval letter with Ref:EPP/172/11/24 is enclosed).

## Data Availability

The data underlying the results of this study are available on *figshare.com* , entitled Project_AI_NNSs DOI: 10.6084/m9.figshare.28270367 ( Shormani, 2025). The project contained two types of data: participants’ responses and ChatGPT responses. Data are available under the terms of the Creative Commons Attribution 4.0 International license (CC-BY 4.0).
