# Supplementary material for: Non-native speakers of English or ChatGPT: Who thinks better?
Source: F1000Res. 2025 Mar 31;14:198. Originally published 2025 Feb 12. [Version 2] doi: 10.12688/f1000research.161306.2 (PMC12503061; doi:10.12688/f1000research.161306.2)
Supplement: Peer review history

# Reviewer report: Reviewer response for version 2

**DOI:** 10.5256/f1000research.179684.r374897

**Published:** 2026-04-15

**Authors:** Osamah Mohammed Alyasiri

**Affiliations:** 1 Al-Furat Al-Awsat Technical University, Karbala, Iraq

1. The conclusions appear to be broader than what the data can support. Given the limited sample size, the use of a single test sentence, and reliance on ChatGPT-3.5, claims such as the human brain “surpasses ChatGPT” or that LLMs cannot be considered theories of language should be moderated. It is recommended to frame the conclusions within the specific scope of the study.
2. The study relies on only one center-embedding sentence as the experimental stimulus. This significantly limits the generalizability of the findings. Incorporating multiple sentences with varying levels of syntactic complexity would improve the robustness and validity of the results.
3. The exclusion of native speakers as a control group weakens the comparative dimension of the study. Including such a group would allow for a more meaningful interpretation of non-native speakers’ performance and strengthen claims related to linguistic competence.
4. The interpretation that ChatGPT’s errors reflect a lack of “linguistic competence” may be theoretically biased. These errors could also be influenced by factors such as prompt design, model limitations, or training data. A more balanced discussion is recommended.
5. The study uses a single prompt format when evaluating ChatGPT. Given the sensitivity of LLMs to prompt design, it is advisable to test multiple prompt formulations to ensure the consistency and reliability of the model’s responses.
6. The analysis is primarily descriptive, relying on percentages and frequencies. The inclusion of inferential statistical tests (e.g., chi-square test) would strengthen the validity of the findings and provide more rigorous support for the conclusions.
7. The paper sometimes conflates theoretical arguments (e.g., generative linguistics, Universal Grammar) with empirical findings. It would be beneficial to clearly separate data-driven results from theoretical interpretations to improve clarity and objectivity.
8. The study is based on ChatGPT-3.5, which is now considered outdated. This limitation should be more explicitly emphasized, as newer models may exhibit significantly different performance. This factor impacts the generalizability of the results.
9. The section discussing ChatGPT’s ability to predict and remember (e.g., “tomorrow” and “yesterday”) appears anecdotal and lacks methodological rigor. It is recommended to either remove this section or clearly frame it as an exploratory observation rather than a core result.
10. Some expressions used in the paper (e.g., “strange,” “nonsense”) are informal and may not be suitable for academic writing. It is recommended to replace them with more formal alternatives such as “inconsistent” or “semantically incoherent.”

Is the work clearly and accurately presented and does it cite the current literature?

Partly

If applicable, is the statistical analysis and its interpretation appropriate?

Partly

Are all the source data underlying the results available to ensure full reproducibility?

No source data required

Is the study design appropriate and is the work technically sound?

Partly

Are the conclusions drawn adequately supported by the results?

No

Are sufficient details of methods and analysis provided to allow replication by others?

Partly

Reviewer Expertise:

AI

I confirm that I have read this submission and believe that I have an appropriate level of expertise to confirm that it is of an acceptable scientific standard, however I have significant reservations, as outlined above.

# Reviewer report: Reviewer response for version 2

**DOI:** 10.5256/f1000research.179684.r374896

**Published:** 2025-05-15

**Authors:** Fawaz Qasem

**Affiliations:** 1 University of Bisha, Bisha, Saudi Arabia

I am very pleased to write a review on this interesting and up-to-date topic.

  The idea of research is informative and has a good theoretical background. All the sections and ideas are well-reported.

 Adapting the framework of Chomsky's generative linguistics gives a strong theoretical foundation for the research paper and the idea of research.

 The results were reported well and the focus of research on center-embedding English constructions was a good idea.

 Addressing both linguistics and cognitive capabilities and sentences’ processing of ChatGPT, AI-powered model, and human’s is productive though exploring cognitive capabilities and the differences between human and AI models require sophisticated tools, experiments, and large data.

 Having enough representative sample of the study would have made the findings and claims stronger and robust.

 Having a recent and advanced version of ChatGPT may lead to different results and night ChatGPT perform better.

 Having a large corpus with a variety of sentences patterns and samples would make the findings more productive and dynamic.

Is the work clearly and accurately presented and does it cite the current literature?

Yes

If applicable, is the statistical analysis and its interpretation appropriate?

Partly

Are all the source data underlying the results available to ensure full reproducibility?

Partly

Is the study design appropriate and is the work technically sound?

Yes

Are the conclusions drawn adequately supported by the results?

Yes

Are sufficient details of methods and analysis provided to allow replication by others?

Partly

Reviewer Expertise:

Linguistics, Applied Linguistics and Acquisition of L2, Psycholinguistics, Sociolinguistics, Corpus Linguistics, educational technology and ESP.

I confirm that I have read this submission and believe that I have an appropriate level of expertise to confirm that it is of an acceptable scientific standard.

# Response

**Published:** 2025-05-15

**Authors:** Mohammed Q. Shormani

**Affiliations:** English Studies, Ibb University, Ibb, Ibb, Yemen

***Dear reviewer,***

***Thank you very much for your constructive review. Thank you also for approving the article in its version 2. Your valuable suggestions are highly appreciated.***

# Reviewer report: Reviewer response for version 2

**DOI:** 10.5256/f1000research.179684.r377338

**Published:** 2025-05-06

**Authors:** FX. Risang Baskara

**Affiliations:** 1 Universitas Sanata Dharma, Depox, Indonesia

**Peer Review Report**

**Summary of the Article**

 This study investigates cognitive processing differences between non-native speakers of English (NNSs) and ChatGPT, specifically examining their ability to interpret center-embedding English constructions. The research recruited 15 advanced non-native English speakers (primarily MA and PhD students with Arabic as their first language) and presented them with a triple center-embedded sentence: "The man that the soldier that the thief slapped deceived died." Participants were asked to identify the actions performed by each entity in the sentence, and the same task was given to ChatGPT-3.5 Turbo.

 The findings indicate that human participants significantly outperformed ChatGPT, with approximately 73.3% of human responses being correct compared to only one correct response (out of three possible actions) from ChatGPT. The study also assessed ChatGPT's grammaticality judgment, finding that it incorrectly deemed the center-embedded sentence ungrammatical, while NNSs recognized it as grammatical. Additional testing revealed ChatGPT's limitations in basic prediction and memory tasks, such as identifying days of the week.

 The author frames these results within generative linguistic theory, arguing that the findings demonstrate the superiority of human language processing capabilities—even for non-native speakers—over Large Language Models. The study concludes that ChatGPT cannot be considered a robust theory of language and that the human brain possesses unique language processing abilities that AI systems have yet to match.

**Is the work clearly and accurately presented and does it cite the current literature?**

**Partly**

 The manuscript has improved considerably through the revision process. The expanded literature review now presents a more balanced perspective by incorporating both supporting viewpoints (Piantadosi, 2023; Ambridge & Blything, 2024) and critical perspectives (Katzir, 2023; Fox & Katzir, 2024; Reiss & Volenec, 2024) on language models. The addition of studies on non-native speakers' processing of complex syntactic structures (Lee & Phillips, 2023; Wu et al., 2020) and LLMs in academic writing (Li et al., 2024; Hwang et al., 2023) helps situate the research within a broader context.

 However, several issues persist:

1. The theoretical framing remains heavily weighted toward generative linguistics, with less comprehensive treatment of alternative perspectives on language processing. While the author has added some counterbalancing views, the integration of these perspectives into the overall theoretical framework is somewhat superficial.
2. The literature on center-embedding specifically in second language acquisition contexts is still limited. Given that the study focuses on NNSs processing center-embedded structures, more targeted literature in this area would strengthen the theoretical grounding.
3. The discussion of ChatGPT's capabilities would benefit from more empirical studies specifically addressing its performance on syntactic parsing tasks, rather than primarily theoretical arguments about LLMs in general.

To address these issues, the authors should:

- Further integrate the newly added literature into the theoretical framework rather than simply mentioning these works
- Include more specific literature on center-embedding in L2 acquisition
- Incorporate more empirical studies on LLMs' performance on complex syntactic parsing tasks

**Is the study design appropriate and is the work technically sound?**

**Partly**

 The authors have clarified several methodological aspects in version 2, but fundamental design limitations remain:

1. **Single test stimulus**: Despite the authors' justification that using one center-embedded sentence maintains consistency in error patterns and avoids redundancy, this remains a significant limitation. A scientifically sound investigation of processing abilities requires multiple test items to establish reliability and account for potential idiosyncrasies in individual sentences.
2. **Sample considerations**: The authors have explained their rationale for using a homogeneous group of 15 advanced NNSs (to minimize variability in language proficiency), but this small, specialized sample limits the generalizability of findings. The authors have acknowledged this limitation, which is appropriate, but the conclusions drawn should be more carefully qualified given this constraint.
3. **Model selection**: The authors have clarified that ChatGPT-3.5 Turbo was used due to resource constraints, and they have acknowledged this as a limitation. This explanation is reasonable, but again requires more careful qualification of the conclusions, as findings may not generalize to more advanced models.
4. **Control considerations**: The authors justify not including a native speaker control group by explaining that their primary aim was to compare NNSs to ChatGPT rather than to native speakers. While this explanation provides clarity about the research focus, it does limit the broader interpretative framework for understanding the findings.

To strengthen the technical soundness:

- Include at least 2-3 additional center-embedded sentences of varying complexity to establish the reliability of findings across different stimuli
- More explicitly qualify conclusions to reflect the limitations of the sample and model selection
- Consider including a pilot comparison with a more advanced LLM (if now accessible) to address whether findings generalize beyond ChatGPT-3.5

**Are sufficient details of methods and analysis provided to allow replication by others?**

**Partly**

 Version 2 includes significant methodological improvements, particularly the addition of section 4.3 (Methods of Analysis) and expanded details about participant characteristics and data collection procedures. However, some gaps remain:

1. **Analysis framework**: While the new section on analysis methods outlines the general approach, it lacks specific details about how responses were coded. For example, what criteria determined whether a response was "correct," "partially correct," or "incorrect"? Were there specific error categories that were identified and analyzed?
2. **ChatGPT testing protocol**: The exact procedure for testing ChatGPT needs more detail. The manuscript specifies that the test was conducted on October 3, 2024, but does not clearly indicate whether multiple attempts were made, how consistency was ensured, or whether different prompting strategies were tried.
3. **Control measures**: While the authors mention that participants were instructed to answer individually, more information about how compliance with this instruction was verified would strengthen methodological rigor.

To improve replicability:

- Provide a more detailed coding scheme for categorizing responses
- Specify the exact prompts used with ChatGPT and whether any variations were attempted
- Explain any measures taken to verify that participants responded independently
- Include the complete protocol for the supplementary test of ChatGPT's ability to predict and remember days of the week

**If applicable, is the statistical analysis and its interpretation appropriate?**

**Not applicable**

 The study primarily uses descriptive statistics (percentages of correct answers) without inferential statistics. Given the qualitative nature of the study and small sample size, this approach is appropriate.

**Are all the source data underlying the results available to ensure full reproducibility?**

**Yes**

 The author has made both the participants' responses and ChatGPT's responses available on Figshare, which supports reproducibility of the analysis. This is a strength of the paper.

**Are the conclusions drawn adequately supported by the results?**

**Partly**

 The authors have moderated some of their conclusions in version 2, but certain claims still extend beyond what the evidence supports:

1. **Generalization from limited data**: The study makes broad claims about human cognitive superiority over AI based on performance on a single center-embedded sentence. While the findings do demonstrate better performance by the NNSs in this specific case, generalizing to all aspects of linguistic processing is not warranted by this limited evidence.
2. **Theoretical implications**: The discussion of competence vs. performance makes interesting points but extends beyond what the current data can support. The theoretical framework involves complex claims about the nature of language and cognition that require more extensive empirical support.
3. **ChatGPT's day-of-the-week task**: This supplementary test is presented as significant evidence of AI memory limitations, but it appears somewhat anecdotal and lacks methodological rigor compared to the main study.

To better align conclusions with evidence:

- Qualify claims about human vs. AI processing to specifically reference the tested phenomena rather than making sweeping generalizations
- Frame theoretical implications as possibilities to be explored in future research rather than established conclusions
- Either strengthen the methodological rigor of the day-of-the-week test or reduce its prominence in the conclusions

**Points that must be addressed to make the article scientifically sound:**

1. **Multiple test stimuli**: Include at least 2-3 additional center-embedded sentences of varying complexity to establish reliability across different stimuli. This is essential for supporting claims about processing capabilities.
2. **Scope qualification**: Explicitly limit conclusions to the specific model tested (ChatGPT-3.5) and the specific linguistic phenomenon examined (center-embedding), acknowledging that findings may not generalize to all aspects of language processing or all LLMs.
3. **Methodological detail**: Provide a more comprehensive coding scheme for response analysis, including specific criteria for categorizing correct and incorrect responses, and more detailed information about the ChatGPT testing protocol.
4. **Theoretical alignment**: Ensure that theoretical claims about linguistic competence, performance, and AI capabilities are appropriately qualified given the limited scope of the evidence.

The authors have made commendable improvements to the manuscript through the revision process, addressing many previous concerns by expanding the literature review, clarifying methodological choices, and adding analysis details. With further attention to the issues outlined above—particularly the need for multiple test stimuli and appropriately scoped conclusions—this study could make a valuable contribution to our understanding of the differences between human and AI language processing capabilities.

Is the work clearly and accurately presented and does it cite the current literature?

Partly

If applicable, is the statistical analysis and its interpretation appropriate?

Not applicable

Are all the source data underlying the results available to ensure full reproducibility?

Yes

Is the study design appropriate and is the work technically sound?

Partly

Are the conclusions drawn adequately supported by the results?

Partly

Are sufficient details of methods and analysis provided to allow replication by others?

Partly

Reviewer Expertise:

- Technology-Enhanced Language Learning (TELL)- Blended Learning Approaches in EFL Contexts- Flipped Classroom Methodologies for Language Acquisition- Artificial Intelligence Applications in Language Education- Computer-Assisted Language Learning (CALL)- Digital Tools and Platforms for EFL Teaching- Innovative Pedagogies in Second Language Acquisition

I confirm that I have read this submission and believe that I have an appropriate level of expertise to confirm that it is of an acceptable scientific standard, however I have significant reservations, as outlined above.

# Response

**Published:** 2025-05-15

**Authors:** Mohammed Q. Shormani

**Affiliations:** English Studies, Ibb University, Ibb, Ibb, Yemen

***Dear reviewer,***

***Thank you very much for your time and efforts in reviewing the article. Thank you also for acknowledging that the study has been improved considerably in version 2. I just wanted to bring to your kind notice that I have made it clear in version 2 that the methodological design, results, and conclusions are limited to this very study, I have also acknowledged all these in the study limitations.***

# Reviewer report: Reviewer response for version 2

**DOI:** 10.5256/f1000research.179684.r374526

**Published:** 2025-04-10

**Authors:** Hui Zong

**Affiliations:** 1 Sichuan University, Chengdu, Sichuan, China

No further comments

Is the work clearly and accurately presented and does it cite the current literature?

Yes

If applicable, is the statistical analysis and its interpretation appropriate?

Not applicable

Are all the source data underlying the results available to ensure full reproducibility?

Yes

Is the study design appropriate and is the work technically sound?

Partly

Are the conclusions drawn adequately supported by the results?

Partly

Are sufficient details of methods and analysis provided to allow replication by others?

Partly

Reviewer Expertise:

medical informatics; artificial intelligence

I confirm that I have read this submission and believe that I have an appropriate level of expertise to confirm that it is of an acceptable scientific standard.

# Response

**Published:** 2025-04-14

**Authors:** Mohammed Q. Shormani

**Affiliations:** English Studies, Ibb University, Ibb, Ibb, Yemen

***Dear reviewer,***

***Thank you very much for your constructive review, which has contributed much to improving the article in form and content. Thank you also for approving our amendments in version 2.***

# Reviewer report: Reviewer response for version 1

**DOI:** 10.5256/f1000research.177317.r367807

**Published:** 2025-03-11

**Authors:** Belle Li

**Affiliations:** 1 Purdue University, West Lafayette, Indiana, USA

**Lit review**

 The paper did a good job to provide a theoretical background on center-embedding structures and their significance in linguistic theory. The literature review effectively traces the history of center-embedding research from Chomsky and Miller (1963) through more recent studies, and clearly positions itself within ongoing debates about whether LLMs can be considered theories of language. However, the paper exhibits an imbalanced literature review, heavily citing work supporting the Chomskyan perspective while giving insufficient attention to alternative views. A more balanced presentation of Piantadosi’s (2023) arguments and similar perspectives is needed to fairly represent the debate. Additionally, the discussion of AI and language models would benefit from more recent empirical studies comparing human and AI language processing, as the paper references primarily theoretical papers about AI limitations rather than the most current empirical research. Despite focusing on non-native speakers, the paper contains minimal discussion of relevant research on second language processing of complex syntactic structures, which is crucial for contextualizing the findings.

**Research Design**

 The use of center-embedded structures is appropriate given their known complexity and processing challenges, with a clearly operationalized research question that captures interesting data points about both human and AI language processing. However, the study’s reliance on only 15 participants, all advanced learners from similar academic backgrounds, significantly limits generalizability. Using only one center-embedded sentence severely restricts the study’s validity. The author acknowledges this limitation but does not sufficiently justify it. Multiple test sentences with varying complexity levels are necessary for robust conclusions. The absence of a native speaker control group makes it impossible to contextualize NNS performance within broader language processing capabilities, a fundamental design flaw that must be addressed. Besides, testing only ChatGPT-3.5, which was not the most advanced model available at the time, limits the study's relevance. The author should test multiple LLMs, including more advanced models, or clearly acknowledge this as a significant limitation.

**Methods & Analysis**

 The test sentence is clearly specified, along with the prompt given to participants and ChatGPT, and the basic procedure for data collection is described. However, the paper lacks crucial details about participant selection criteria, specific language backgrounds, and how their “advanced” status was determined. These details are essential for replication. The informal WhatsApp-based data collection raises methodological concerns that must be addressed, including whether participants were monitored during response formulation, whether they could consult resources or other individuals, the exact instructions given beyond the brief prompt shown, and how much time was allowed for responses. The paper lacks a systematic framework for analyzing responses, where a clearer coding scheme for categorizing correct and incorrect answers would improve reproducibility. Additionally, the paper does not specify how many times ChatGPT was prompted or whether different prompting strategies were attempted, which are crucial details for reproducible AI testing.

**Findings**

 The finding that the tested non-native speakers outperformed ChatGPT-3.5 on the specific center-embedded structure is directly supported by the data, as is the observation that ChatGPT made errors in grammaticality judgment. However, the paper makes broad claims about LLMs as theories of language based on limited evidence. These conclusions must be qualified to acknowledge that testing was limited to one model (ChatGPT-3.5), only one linguistic phenomenon was tested, and the specific limitations identified may not generalize to all aspects of language processing. The discussion of competence vs. performance makes interesting points but extends beyond what the current data can support. The author must either gather additional evidence or moderate claims about the theoretical implications. The section on ChatGPT’s inability to predict days of the week is presented as significant evidence of memory limitations but seems tangential and anecdotal rather than systematically investigated. This should either be developed with more rigorous methods or reduced in prominence.

 To make the article scientifically sound, the methodology must be expanded to include multiple test sentences with varying complexity levels, test performance across a range of center-embedded structures, and provide complete information about participant selection and characteristics. Experimental controls should be improved by adding a native speaker control group for proper comparison, implementing and describing controls for ensuring independent responses, and detailing exact protocols for both human and AI testing. The theoretical framework needs better balance by presenting alternative perspectives on language models more thoroughly, acknowledging limitations in generalizing from one specific linguistic phenomenon, and considering how technological improvements might affect conclusions. Finally, the scope should be better limited by clearly acknowledging the preliminary nature of findings given sample limitations, restricting conclusions to specifically what was tested rather than broad claims, and discussing how future research might address current limitations.

Is the work clearly and accurately presented and does it cite the current literature?

Partly

If applicable, is the statistical analysis and its interpretation appropriate?

Not applicable

Are all the source data underlying the results available to ensure full reproducibility?

Yes

Is the study design appropriate and is the work technically sound?

Partly

Are the conclusions drawn adequately supported by the results?

Partly

Are sufficient details of methods and analysis provided to allow replication by others?

Partly

Reviewer Expertise:

AI in language learning and teaching

I confirm that I have read this submission and believe that I have an appropriate level of expertise to confirm that it is of an acceptable scientific standard, however I have significant reservations, as outlined above.

# Response

**Published:** 2025-03-12

**Authors:** Mohammed Q. Shormani

**Affiliations:** English Studies, Ibb University, Ibb, Ibb, Yemen

Dear reviewer,

 Thank you very much for the time and efforts you exerted in reviewing the article. Thank you also for your insightful comments and suggestions. Our responses are indicated as *text.*

 Lit review

 A more balanced presentation of Piantadosi’s (2023) arguments and similar perspectives is needed to fairly represent the debate. Additionally, the discussion of AI and language models would benefit from more recent empirical studies comparing human and AI language processing, as the paper references primarily theoretical papers about AI limitations rather than the most current empirical research. Despite focusing on non-native speakers, the paper contains minimal discussion of relevant research on second language processing of complex syntactic structures, which is crucial for contextualizing the findings.

*Response* *:*

*Thank you for your insightful comment. We have expanded our literature review to provide a more balanced discussion of Piantadosi (2023), Fox and Katzir (2024) and* *Reiss and Volenec (2024)* *,* *thus incorporating both supporting and opposing viewpoints.* *Thank you also for suggesting adding* *discussion of relevant research on second language processing of complex syntactic structures, which is crucial for contextualizing the findings. Thus, we incorporated relevant studies including Wu et al. (2020), Lee and Phillips (2023). We also added some studies concerning enhancing the research and academic writing abilities of non-native speakers of English through LLMs including Li et al. (2024) and* *Hwang et al. (2024).*

 Research design

 However, the study’s reliance on only 15 participants, all advanced learners from similar academic backgrounds, significantly limits generalizability. Using only one center-embedded sentence severely restricts the study’s validity. The author acknowledges this limitation but does not sufficiently justify it. Multiple test sentences with varying complexity levels are necessary for robust conclusions. The absence of a native speaker control group makes it impossible to contextualize NNS performance within broader language processing capabilities, a fundamental design flaw that must be addressed.

*Response* :

*Thank you for your insightful comment. We agree with you regarding the sample size and the use of a single center-embedded sentence. However, our concern was to focus on a homogeneous group of advanced NNSs of English to minimize variability in language proficiency, which allowed for a more controlled investigation of their performance relative to ChatGPT. While a larger and more diverse sample would enhance generalizability, our primary aim was to explore how advanced NN learners process complex syntactic structures rather than to make broad generalizations about all non-native speakers of English. Concerning the use of a single center-embedded sentence, and for the scope of this study, we felt that focusing on a single sentence allowed us to draw clear comparisons and avoid unnecessary* *complexity. As outlined in the paper, we selected a single sentence for two key reasons: i) consistency in error patterns: We aimed to ensure that if human participants made errors in one sentence, they would likely make the same errors in similar sentences (of the same type, i.e. center-embedding), and the same would apply to ChatGPT. By focusing on a single sentence, we could effectively examine error patterns without introducing variability that might arise from different sentence structures, and ii) avoiding redundancy **:** Using a single sentence allowed us to focus on a specific type of linguistic phenomenon, reducing redundancy and ensuring that the data were not overwhelmed by repeated interpretations of the same errors, whether made by human participants or ChatGPT. As for the absence of* *native speaker control group*, *our primary focus was to examine how advanced NNSs process center-embedding structures in comparison to ChatGPT, rather than to compare their performance to native speakers. By keeping the participant pool homogeneous, we aimed to minimize variability in proficiency levels and maintain a controlled investigation.*

 Besides, testing only ChatGPT-3.5, which was not the most advanced model available at the time, limits the study's relevance. The author should test multiple LLMs, including more advanced models, or clearly acknowledge this as a significant limitation.

*Response:*

*Thank you for your insightful comment. We agree that more advanced models such as ChatGPT-4 and Grok-3 are now available and have stronger reasoning capabilities. However, as noted in the article, we utilized ChatGPT-3.5 Turbo because it was the only free version accessible to us at the time of the study. Due to resource constraints and the timing of our research, we were unable to access the more advanced models for comparison. We have also acknowledged this as one of the important limitations of the study.*

 Methods and analysis

 However, the paper lacks crucial details about participant selection criteria, specific language backgrounds, and how their “advanced” status was determined. These details are essential for replication. The informal WhatsApp-based data collection raises methodological concerns that must be addressed, including whether participants were monitored during response formulation, whether they could consult resources or other individuals, the exact instructions given beyond the brief prompt shown, and how much time was allowed for responses.

*Response* :

*Thank you for your insightful comment. We have incorporated details concerning methodological issues such as participant selection criteria, language backgrounds, WhatsApp-based data collection, among other related aspects.*

 The paper lacks a systematic framework for analyzing responses, where a clearer coding scheme for categorizing correct and incorrect answers would improve reproducibility. Additionally, the paper does not specify how many times ChatGPT was prompted or whether different prompting strategies were attempted, which are crucial details for reproducible AI testing.

*Response:*

*Thank you for your insightful comment. We have added a new section (* *4.3. Methods of analysis) in which we outlined the framework and approaches to analyzing both NNSs and ChatGPT’s responses.*

 Findings

 However, the paper makes broad claims about LLMs as theories of language based on limited evidence. These conclusions must be qualified to acknowledge that testing was limited to one model (ChatGPT-3.5), only one linguistic phenomenon was tested, and the specific limitations identified may not generalize to all aspects of language processing. The discussion of competence vs. performance makes interesting points but extends beyond what the current data can support. The author must either gather additional evidence or moderate claims about the theoretical implications. The section on ChatGPT’s inability to predict days of the week is presented as significant evidence of memory limitations but seems tangential and anecdotal rather than systematically investigated. This should either be developed with more rigorous methods or reduced in prominence.

*Response:*

*Thank you for your insightful comment. In addition to what has been stated in the manuscript, we have also added that the participants were, in fact, instructed to answer the study question individually. They were also explicitly informed that their responses should reflect their own understanding of the center-embedding sentence. Regarding the idea that “tangential and anecdotal” claims about ChatGPT’s inability to state the date and name of “yesterday” and “tomorrow”, we actually meant that this example illustrates **limitations in ChatGPT’s handling of structured, rule-based knowledge**, while Google “knows” it, but we do not overstate its significance in relation to broader linguistic competence.*

# Reviewer report: Reviewer response for version 1

**DOI:** 10.5256/f1000research.177317.r366335

**Published:** 2025-03-11

**Authors:** Hui Zong

**Affiliations:** 1 Sichuan University, Chengdu, Sichuan, China

This research investigates whether non-native English speakers or ChatGPT exhibit better cognitive processing abilities, specifically in understanding and interpreting center-embedded English constructions. The study involved 15 non-native English speakers and compared their performance against ChatGPT’s ability to predict and remember. The results indicate that the human brain remains superior to large language models like ChatGPT, even for non-native English speakers. Here are my comments:

 1. Were all 15 participants non-native English speakers? What were their native languages? It is recommended to specify this information in the paper.

 2. Was ChatGPT accessed via the official client or third-party software? The experiment was conducted through WhatsApp and the ChatGPT client. Please include details on when the experiment took place.

 3. In the method section, the study tested only one question, which may impact the validity of the conclusion.

 4. The current title may not fully reflect the scope of the research. It is suggested to revise the title to be more specific.

 5. The study only uses ChatGPT-3.5 Turbo, lacking comparisons with other large language models. More advanced models, such as ChatGPT-4o and Grok-3, are now available and have stronger reasoning capabilities. If possible, the authors are encouraged to include comparisons with these models.

 6. Please consider adding the following reference:

 Exploring the potential of artificial intelligence to enhance the writing of English academic papers by non-native English-speaking medical students - the educational application of ChatGPT(Refer 1)

Is the work clearly and accurately presented and does it cite the current literature?

Yes

If applicable, is the statistical analysis and its interpretation appropriate?

Not applicable

Are all the source data underlying the results available to ensure full reproducibility?

Yes

Is the study design appropriate and is the work technically sound?

Partly

Are the conclusions drawn adequately supported by the results?

Partly

Are sufficient details of methods and analysis provided to allow replication by others?

Partly

Reviewer Expertise:

medical informatics; artificial intelligence

I confirm that I have read this submission and believe that I have an appropriate level of expertise to confirm that it is of an acceptable scientific standard, however I have significant reservations, as outlined above.

## References

[1] : Exploring the potential of artificial intelligence to enhance the writing of english academic papers by non-native english-speaking medical students - the educational application of ChatGPT. *BMC Med Educ* .2024;24(1) : 10.1186/s12909-024-05738-y 736 10.1186/s12909-024-05738-y 38982429 PMC11232216

# Response

**Published:** 2025-03-12

**Authors:** Mohammed Q. Shormani

**Affiliations:** English Studies, Ibb University, Ibb, Ibb, Yemen

Dear reviewer,

 Thank you very much for the time and efforts you exerted in reviewing the article. Thank you also for your insightful comments and suggestions. Our responses are indicated as *text.*

 1. Were all 15 participants non-native English speakers? What were their native languages? It is recommended to specify this information in the paper.

*Response*:

*Thank you for this valuable comment. The participants' L1 is Arabic; we have pinpointed this in the article in addition to outlining the L2 English courses they have studded in their study journey.*

 2. Was ChatGPT accessed via the official client or third-party software? The experiment was conducted through WhatsApp and the ChatGPT client. Please include details on when the experiment took place.

*Response:*

*Thank you for your valuable comment. We have made it clear that we accessed ChatGPT through the official ChatGPT client, which ensured that the model used was the standard version available to users at the time of the study. we have also pointed out that the study data (question) was sent to the participants on November 7, 2024.*

 3. In the method section, the study tested only one question, which may impact the validity of the conclusion.

*Response:*

*Thank you for your valuable feedback. We understand your concern regarding the use of only one center-embedding sentence in the study. We have also acknowledged this as one of the limitations of the study in the sense of involving other constructions such as those involving DP islands, multiple wh-questions, anaphora, and weak/strong crossovers (different phenomena). However, for the scope of this study, we felt that focusing on a single sentence allowed us to draw clear comparisons and avoid unnecessary complexity. As outlined in the paper we selected a single sentence for two key reasons: i) consistency in error patterns **:** We aimed to ensure that if human participants made errors in one sentence, they would likely make the same errors in similar sentences (of the same type, i.e. center-embedding), and the same would apply to ChatGPT. By focusing on a single sentence, we could effectively examine error patterns without introducing variability that might arise from different sentence structures, and ii) avoiding redundancy **:** Using a single sentence allowed us to focus on a specific type of linguistic phenomenon, reducing redundancy and ensuring that the data were not overwhelmed by repeated interpretations of the same errors, whether made by human participants or ChatGPT.*

 4. The current title may not fully reflect the scope of the research. It is suggested to revise the title to be more specific.

*Response:*

*Thank you for your feedback regarding the title. After considering your suggestion, we have decided to retain the original title, Non-native speakers of English or ChatGPT: Who thinks better?, as it directly reflects the central focus of the study—comparing the cognitive performance and language processing abilities of non-native English speakers and ChatGPT. The term "thinking" could involve both the ability of processing/interpreting and remembrance, which was aimed at. Thus, we believe that such phrasing captures the essence of the study in a way that is both engaging and informative for the reader, highlighting the core questions of our study.*

 5. The study only uses ChatGPT-3.5 Turbo, lacking comparisons with other large language models. More advanced models, such as ChatGPT-4o and Grok-3, are now available and have stronger reasoning capabilities. If possible, the authors are encouraged to include comparisons with these models.

*Response:*

*Thank you for your insightful comment. We acknowledge that more advanced models such as ChatGPT-4 and Grok-3 are now available and have stronger reasoning capabilities. However, as noted in the article, we utilized ChatGPT-3.5 Turbo because it was the only free version accessible to us at the time of the study. Due to resource constraints and the timing of our research, we were unable to access the more advanced models for comparison. We have also acknowledged this as one of the limitations of the study.*

 6. Please consider adding the following reference:

 Exploring the potential of artificial intelligence to enhance the writing of English academic papers by non-native English-speaking medical students - the educational application of ChatGPT(Refer 1)

*Response:*

*Thank you very much for suggesting adding this interesting reference; we have added it in addition to some others including Hwang et al. (2023).*
